# ITGAM-mediated macrophages contribute to basement membrane damage in diabetic nephropathy and atherosclerosis

**DOI:** 10.1186/s12882-024-03505-1

**Published:** 2024-02-27

**Authors:** Yude Lou, Peng Hui Li, Xiao Qi Liu, Tian Xiang Wang, Yi Lan Liu, Chen Chen Chen, Kun Ling Ma

**Affiliations:** 1 Department of Nephrology, the Second Affiliated Hospital, School of Medicine, Zhejiang University, Hangzhou, 310009 China; 2 Institute of Immunology, School of Medicine, Zhejiang University, Hangzhou, 310058 China; 3 Department of Basic Medicine Sciences, School of Medicine, Zhejiang University, Hangzhou, 310058 China

**Keywords:** Atherosclerosis, Basement membrane, Diabetic nephropathy, Integrin subunit alpha M

## Abstract

**Background:**

Diabetic nephropathy (DN) and atherosclerosis (AS) are prevalent and severe complications associated with diabetes, exhibiting lesions in the basement membrane, an essential component found within the glomerulus, tubules, and arteries. These lesions contribute significantly to the progression of both diseases, however, the precise underlying mechanisms, as well as any potential shared pathogenic processes between them, remain elusive.

**Methods:**

Our study analyzed transcriptomic profiles from DN and AS patients, sourced from the Gene Expression Omnibus database. A combination of integrated bioinformatics approaches and machine learning models were deployed to identify crucial genes connected to basement membrane lesions in both conditions. The role of integrin subunit alpha M (*ITGAM*) was further explored using immune infiltration analysis and genetic correlation studies. Single-cell sequencing analysis was employed to delineate the expression of *ITGAM* across different cell types within DN and AS tissues.

**Results:**

Our analyses identified *ITGAM* as a key gene involved in basement membrane alterations and revealed its primary expression within macrophages in both DN and AS. ITGAM was significantly correlated with tissue immune infiltration within these diseases. Furthermore, the expression of genes encoding core components of the basement membrane was influenced by the expression level of *ITGAM.*

**Conclusion:**

Our findings suggest that macrophages may contribute to basement membrane lesions in DN and AS through the action of *ITGAM*. Moreover, therapeutic strategies that target ITGAM may offer potential avenues to mitigate basement membrane lesions in these two diabetes-related complications.

**Supplementary Information:**

The online version contains supplementary material available at 10.1186/s12882-024-03505-1.

## Introduction

Diabetic nephropathy (DN) represents a prevalent and serious complication of diabetes mellitus (DM), contributing to heightened mortality rates among individuals affected by diabetes [1]. Hyperglycemia is considered to be the most important risk factor for the development of proteinuria and even end-stage renal disease (ESRD) in DN. As a microvascular complication of DM, the earliest consistent change in DN is thickening of the glomerular basement membrane (GBM). Other glomerular pathological alterations include loss of podocytes with effacement of foot processes, mesangial matrix expansion, and loss of endothelial fenestrations [2]. It has been suggested that tubulointerstitium injury (interstitial fibrosis and tubular atrophy) in DN can even precede glomerulopathy [3]. Tubular basement membrane (TBM) thickness is also of high predictive value in patients who progress to ESRD [4]. Clinically, proteinuria is the main manifestation of DN and its onset is often later than GBM thickening. By the time patients develop microproteinuria, the structural alterations in the kidney are typically quite severe [5]. Despite these findings, the relationship between these structural changes and the onset of proteinuria remains inadequately understood. Therefore, to fully appreciate the significance of these structural changes, it is vital to explore and elucidate the pathophysiological mechanisms underlying these alterations attributed to DN.

Diabetic patients not only face the risk of microvascular complications, but also frequently exhibit concomitant atherosclerosis (AS). Increasingly, elevated blood glucose levels are being recognized as an independent risk factor for both all-cause and cardiovascular mortality [6, 7]. The pathogenesis of atherosclerosis involves initial damage to the intima, followed by degradation and subsequent remodeling of the basement membrane. This series of changes opens up the pathway for lipid infiltration and the migration of monocytes and lymphocytes [8, 9]. Remarkably, lesions to the basement membrane have been observed to significantly influence both the onset and progression of DN and atherosclerosis.

Integrins, a crucial family of obligate heterodimers, are instrumental in enabling cell-cell and cell-extracellular matrix adhesion. This family is comprised of alpha and beta subunits. One such integrin, the integrin subunit alpha M (ITGAM), is encoded by the ITGAM gene and forms a complex with the integrin subunit beta 2 chain, thus creating a receptor commonly known as macrophage receptor-1 (MAC-1). ITGAM not only acts as a biomarker for monocyte-macrophage cells but it is also implicated in cell adhesion, chemotaxis, and migration [10]. Abdominal aortic aneurysms show a high expression of ITGAM, indicating its role in mediating macrophage adhesion and transendothelial migration, which in turn leads to macrophage infiltration and arterial inflammation [11]. Additionally, ITGAM is postulated to mediate hypertension-induced cardiac remodeling through the regulation of macrophage infiltration and polarization [12]. These pieces of evidence point toward the potential role of ITGAM in cardiovascular disease by mediating macrophage infiltration. However, its role in DN and AS has yet to be fully elucidated.

This study aimed to elucidate shared molecular mechanisms underlying basement membrane lesions in DN and AS. We harnessed multiple datasets derived from the Gene Expression Omnibus (GEO) database, employing a combination of sophisticated bioinformatics techniques and machine learning methodologies. Our findings highlighted *ITGAM* as a critical gene implicated in basement membrane damage of the glomerulus and tubules in DN, as well as the plaques in AS. We further observed that heightened *ITGAM* expression was associated with increased infiltration of immunocytes into affected tissue. Moreover, the degree of *ITGAM* expression was found to significantly influence the transcriptional activity of genes encoding pivotal components of the basement membrane. Analyses of single-cell sequencing data from DN and AS lesion samples revealed ITGAM to be predominantly expressed in infiltrating macrophages within the disease-affected tissue. Drawing on existing research, our findings provide a possibility that macrophages may contribute to the pathophysiological events causing basement membrane damage in DN and AS, with this involvement mediated by ITGAM.

## Materials and methods

### Data source and processing

The datasets GSE96804, GSE104948, GSE104954, GSE30529, GSE47184, GSE100927, GSE195799, and GSE184073, alongside their associated information, were sourced from the GEO database, extracted in the MINiML format. Upon procurement, the data from unnormalized datasets underwent log2 transformation. In instances where datasets were not standardized, normalization was executed utilizing the normalize.quantiles function provided by the preprocessCore package within R software (version 4.1.2). Subsequently, Probe IDs were transposed to gene symbols in accordance with platform annotations. Where multiple probes corresponded to a singular gene, an average was computed to generate a solitary value representative of that gene. To mitigate batch effects within the same dataset or platform, the removeBatchEffect function, available in the limma package of R software, was employed. During the analysis of data from disparate datasets, an initial step was taken to extract a shared set of gene symbols. Following this, different datasets or platforms were considered as separate batches, and batch effects were adjusted using the removeBatchEffect function. The investigation of mRNA differential expression was conducted using the limma package of the R software. To control for false positives, the adjusted *p* values within GEO were subjected to analysis. A criterion for significant differential expression of mRNA was established as an adjusted *p* value below 0.05.

We conducted an analysis of data derived from multiple sources to gain insights into different aspects of DN. Data from the GSE96804 dataset, containing the glomerular transcriptome of 41 DN samples and 20 unaffected portions of tumor nephrectomy samples, was investigated. The GSE104948 dataset offered glomerular transcriptomes derived from subjects of the European Renal cDNA Bank as well as living donors. Within this dataset, specific samples were selected from two distinct platforms: GPL24120 and GPL22945. Five DN samples and three healthy donors from the GPL24120 platform, alongside seven DN samples and 18 healthy donors from the GPL22945 platform, were incorporated into the analysis.

In studying the renal tubulointerstitium, we selected the complete RNA expression data from the GSE30529 dataset. This dataset features tubule samples from 10 DN patients and 12 healthy controls. Additional samples were chosen from the GSE47184 dataset (specifically, 11 DN samples and four cadaveric donors from the GPL14663 platform). Within the GSE104954 dataset, which includes two sequencing platforms (GPL24120 and GPL22945), we opted for a selection of tubulointerstitium samples from both DN patients and healthy donors. Ten DN samples and three healthy donors from the GPL24120 platform, plus seven DN samples and 18 healthy donors from the GPL22945 platform, were included for further analysis of the tubulointerstitium.

The GSE100927 dataset, encompassing RNA expression data of human peripheral arteries from carotid, femoral, and infra-popliteal territories in atherosclerotic and control tissue, was wholly included in our study. This dataset comprises 69 AS samples and 35 controls.

The GSE195799 dataset, containing single-cell transcriptome data of CD45-enriched kidney immune cells from control and diabetic OVE26 mice, was also utilized. In this case, the diabetic OVE26 mouse sample was selected for a single-cell analysis.

Lastly, we sourced data from the GSE184073 dataset, which includes single-cell RNA sequencing data of human coronary plaques. Specifically, the GBM5577200 sample, diagnosed with acute coronary syndrome, was selected for subsequent study.

We obtained proteomic data through the ProteomeXchange database (https://proteomecentral.proteomexchange.org/). The dataset PXD041367 was selected, which encompasses the proteome of human monocytes from samples of atherosclerosis related and non-related to chronic kidney disease (CKD). Quantitative and qualitative proteomic analyses were conducted using nano liquid chromatography-tandem mass spectrometry (nano LC-MS/MS) to explore the global proteomic alterations in monocytes, which were isolated from the peripheral blood of patients by immunomagnetic separation. These cells were isolated from patients at various stages of atherosclerosis, both related and non-related to CKD.

### Weighted gene co-expression network analysis

In our study, we utilized the Sangerbox clinical bioinformatics analysis platform to perform a weighted gene co-expression network analysis (WGCNA) [13]. The process began with the calculation of the median absolute deviation (MAD) for each gene using the gene expression spectrum. We subsequently eliminated the lower 50% of genes based on the smallest MAD values. Outliers were identified and removed using the WGCNA package in R software, employing the “goodSamplesGenes” function. We proceeded to construct a scale-free co-expression network via WGCNA. This entailed applying the Pearson’s correlation matrices and average linkage method for all pairwise genes. A weighted adjacency matrix was constructed using the power function A_mn = |C_mn|^β (where C_mn is the Pearson’s correlation between gene_m and gene_n, and A_mn represents the adjacency between gene_m and gene_n). Here, β serves as a soft-thresholding parameter that accentuates strong gene correlations and devalues weaker ones. In our analysis, we selected a power of 5. Following this, the adjacency was transformed into a topological overlap matrix (TOM). The TOM quantifies the network connectivity of a gene, defined as the total of its adjacency with all other genes relative to the network gene ratio. We then computed the corresponding dissimilarity, given by 1-TOM. To classify genes with similar expression profiles into modules, we utilized average linkage hierarchical clustering, based on the TOM-dissimilarity measure. This procedure required a minimum size (gene group) of 30 for the gene dendrogram and was performed with a sensitivity setting of three. Further module analysis involved calculating the dissimilarity of module eigengenes, setting a cut-off line for the module dendrogram, and merging specific modules. We also merged any modules with a distance less than 0.25. In the final co-expression modules obtained, we identified the gray module as containing genes that could not be grouped into any module. The hub genes were chosen from the four modules showing the highest correlation, with the selection criteria detailed in the Results section.

### Gene ontology (GO) enrichment analysis

For the enrichment analysis, we employed the R 3.6.3 software with the clusterProfiler package (version 4.2.2). The ID conversion was performed utilizing the “org.Hs.eg.db” package (version 3.14.0). To compute the z-score, the “GOplot” package (version 1.0.2) was used [14, 15].

### Support vector machines-recursive feature elimination (SVM-RFE) algorithm

The SVM-RFE algorithm systematically ranks features recursively, effectively minimizing the risk of model overfitting [16–18]. We utilized the e1071 package in R, incorporating 10-fold cross-validation approach, which was meticulously set up using a corresponding seed to ensure reproducibility and to mitigate potential biases in model training. This approach allowed systematically rank features, thus minimizing the risk of overfitting, which is a common challenge in machine learning models. In this study, the genes identified were utilized as features for the SVM-RFE model, with the average model predictive accuracy/error across different feature quantities being determined via 10-fold cross-validation. The optimal number of genes (denoted as N), was ascertained by choosing the quantity of features corresponding to the highest accuracy and the lowest error of the model. Following the feature ranking provided by the model, the top N genes were screened to constitute a gene set. The common genes identified within the gene sets predicted by the respective models for DN glomerulus, DN tubulointerstitium, and AS, are considered to be robust features, indicating their potential critical roles in the corresponding biological processes. Consequently, these genes warrant further investigation. Using this algorithm, we were able to identify hub basement membrane-related genes.

### Random forest (RF) algorithm

The RF algorithm, a supervised machine learning method, leverages decision tree algorithms to solve regression and classification problems. In the RF model, applied via the randomForest package (version 4.7–1.1) and varSelRF package (version 0.7–8) in R, we focused on quantifying feature importance using the mean decrease in the Gini index, a method effective in reducing bias inherent in feature selection [19]. The model was trained with a predefined number of trees (*N* = 750), a decision made after assessing model performance with a higher initial number of trees (*N* = 1000). This process of tuning the number of trees was crucial to balance model accuracy and computational efficiency, thus preventing overfitting. To further ensure the generalizability of our findings, we performed cross-validation in our RF model as well, including a repeated measure. This step is vital in assessing the stability and reliability of our model’s predictions across different subsets of the data. In this study, the genes identified were utilized as variables for the RF model. Following five-fold cross-validation, the optimal number of genes (denoted as N), was determined based on the corresponding number of variables that resulted in a low mean prediction error. The top N genes were then selected in accordance with their ranking of variable importance (assessed by the mean decrease in the Gini index), as the best predictors. Due to the inherent randomness of the RF model, its predictive outcomes are challenging to interpret. The predictive outcomes are also susceptible to the influence of noise within the dataset. Consequently, we employed it solely in AS, serving as a validation for the SVM-RFE model, thereby enhancing the reliability of results. We assessed the predictive significance of these hub genes by creating a receiver operating characteristic (ROC) curve using the pROC package in R software [20].

### Evaluation of immune infiltration

We used the estimation of stromal and immune cells in malignant tumor tissues using expression data (ESTIMATE) algorithm to calculate tumor purity [21]. This study further examined the comprehensive profile of immune infiltration in DN and AS samples using the ESTIMATE algorithm. The single-sample gene set enrichment analysis (ssGSEA), an extension of the GSEA method, permits the calculation of an enrichment score. This score symbolizes the absolute degree of a gene set’s enrichment in each sample within a given dataset [22]. We deployed ssGSEA to analyze further the infiltration levels of diverse immune cells, contingent on whether *ITGAM* was expressed at high or low levels.

### Single-cell RNA sequencing data analysis

The single-cell RNA sequencing data was derived from the gene count matrix of GSE195799 and GSE184073, which were acquired from the GEO database. Specifically, the barcode data, gene feature data, and gene count matrix data of GSE184073, all preprocessed by Cellranger (10X Genomics), were downloaded. The analysis was conducted using the R statistical software, version 4.1.2, leveraging the capabilities of the Seurat package (version 4.3.0) [23].

### Statistical analysis and visualization

Statistical analyses were performed using the R statistical software. The data were presented as the mean value ± the standard error of the mean (SEM). Depending on the data distribution and variance, different tests were employed for two-group comparisons: Student’s *t*-test, Welch’s *t*-test, or the Wilcoxon rank sum test. In addition, Spearman’s correlation analysis was used to determine correlations between two datasets, providing the correlation coefficient. A *p* value of less than 0.05 was deemed to indicate statistical significance. All data visualization tasks were also completed utilizing the R software.

## Results

### Identification of key basement membrane-related genes in DN glomerulus and tubulointerstitium

**Fig. 1:**
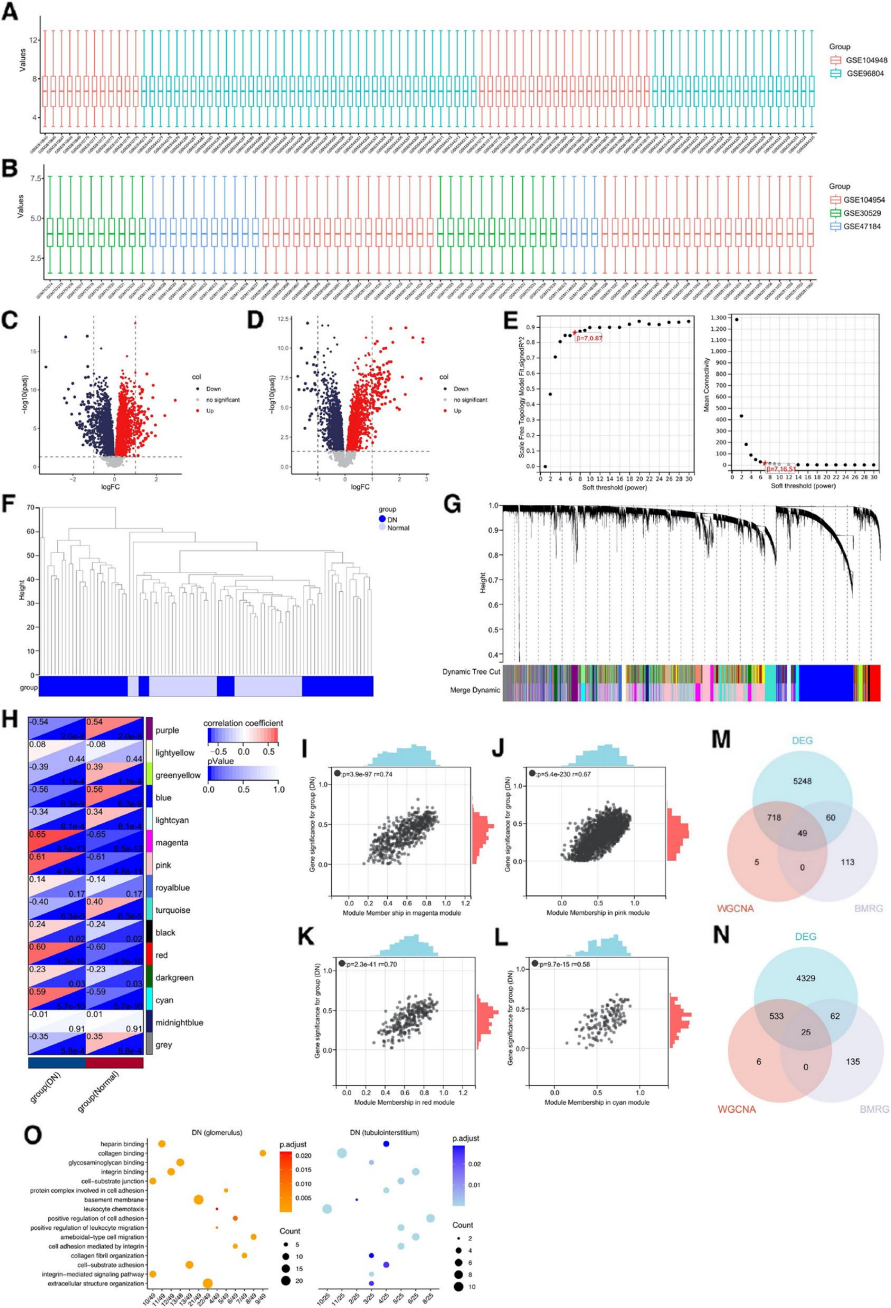
Identification of key basement membrane-related genes in diabetic nephropathy glomerulus and tubulointerstitium. **A** Merging, standardization, and batch effect removal of GSE104948 and GSE96804 datasets. The merged dataset includes 53 DN glomerulus and 41 normal control samples. **B** Merging, standardization, and batch effect removal of GSE104954, GSE30529, and GSE47184 datasets. The merged dataset contains 38 DN tubulointerstitium and 37 normal control samples. **C**, **D** Generation of volcano plots of DEGs in DN glomerulus and tubulointerstitium. Genes with an adjusted *p* value less than 0.05 were deemed differentially expressed. **E** Optimization of the soft threshold β, set to 7. **F** Hierarchical clustering of GSE104948 and GSE96804 samples based on gene expression levels. **G** Identification of co-expression modules in DN glomerulus with the following parameters: minimum module size: 30; sensitivity: 3; module merge threshold: 0.25. **H** Correlation between the 15 identified co-expression modules and DN (glomerular group). Four modules (magenta, pink, red, and cyan) showed the highest DN correlation. **I**-**L** Scatter plots illustrating correlation between genes (module membership) in the top four modules and DN (glomerular group). **M**, **N** Intersection of DEGs, genes screened by WGCNA, and BMRGs in both DN glomerular and tubulointerstitial groups. **O** GO enrichment analysis of intersected genes in DN glomerulus and tubulointerstitium

To understand the gene expression profiles within the glomerulus and tubulointerstitium in the context of DN, selected samples from GSE96804 and GSE104948 were aggregated for a combined analysis. This examination incorporated 53 DN samples and 41 healthy control samples (Fig. 1A). Additionally, chosen samples from GSE30529, GSE47184, and GSE104954, which totaled 38 DN samples and 37 healthy controls, were combined for a comprehensive study on the tubulointerstitium (Fig. 1B). Utilizing the limma package, a differential gene expression analysis was carried out between DN and healthy control samples within the glomerulus dataset. This resulted in the identification of 3018 significantly upregulated and 3057 significantly downregulated genes (Fig. 1C). A similar analysis within the DN tubulointerstitium dataset revealed 2102 upregulated and 2847 downregulated genes (Fig. 1D). To pinpoint genes demonstrating the highest phenotype correlation, a WGCNA was performed. Hierarchical clustering of DN glomerulus samples, based on gene expression levels, revealed no outliers (Fig. 1F). Subsequently, the optimal soft threshold β was determined as 7 (Fig. 1E), with a minimum gene module size of 30 and sensitivity set to 3, allowing the categorization of genes with similar expression profiles into modules. The dissimilarity of module characteristic genes was calculated and modules exhibiting a distance of less than 0.25 apart were amalgamated by cutting line in the module dendrogram. This process yielded 15 co-expression modules (Fig. 1G). Correlation analysis of the 15 co-expression modules and DN revealed the magenta, pink, red, and cyan modules as having the highest correlation (Fig. 1H). Genes (module membership) within these four modules also demonstrated a high degree of correlation with DN (Fig. 1I-L). Applying a screening criterion of a MM-GS correlation coefficient greater than 0.7, 772 genes were selected from these four modules for subsequent analysis (Supplementary Table 1). A comparable analysis was carried out on the tubulointerstitium samples (Supplementary Fig. 1A-I), resulting in the identification of 13 co-expression modules. Among these, the pink, dark orange, light cyan, and dark green modules had the highest correlation with DN. Again, using the same screening criterion, 564 genes were selected from these four modules for further investigation (Supplementary Table 2).

Upon reviewing the available literature, we identified 222 basement membrane-related genes (BMRGs) [24]. Through an intersecting analysis of differential expressed genes (DEGs), genes selected by WGCNA, and BMRGs, we determined 49 hub basement membrane-related genes in the DN glomerulus, as well as 25 genes in the DN tubulointerstitium, as depicted in Fig. 1M and N. To further elucidate the functional roles of these hub genes, we performed GO enrichment analysis. Our findings suggest that these genes are predominantly implicated in chemotaxis, cell migration, and adhesion, with particular emphasis on leukocyte interactions (Fig. 1O).

### Identification of key basement membrane-related genes in atherosclerosis

**Fig. 2:**
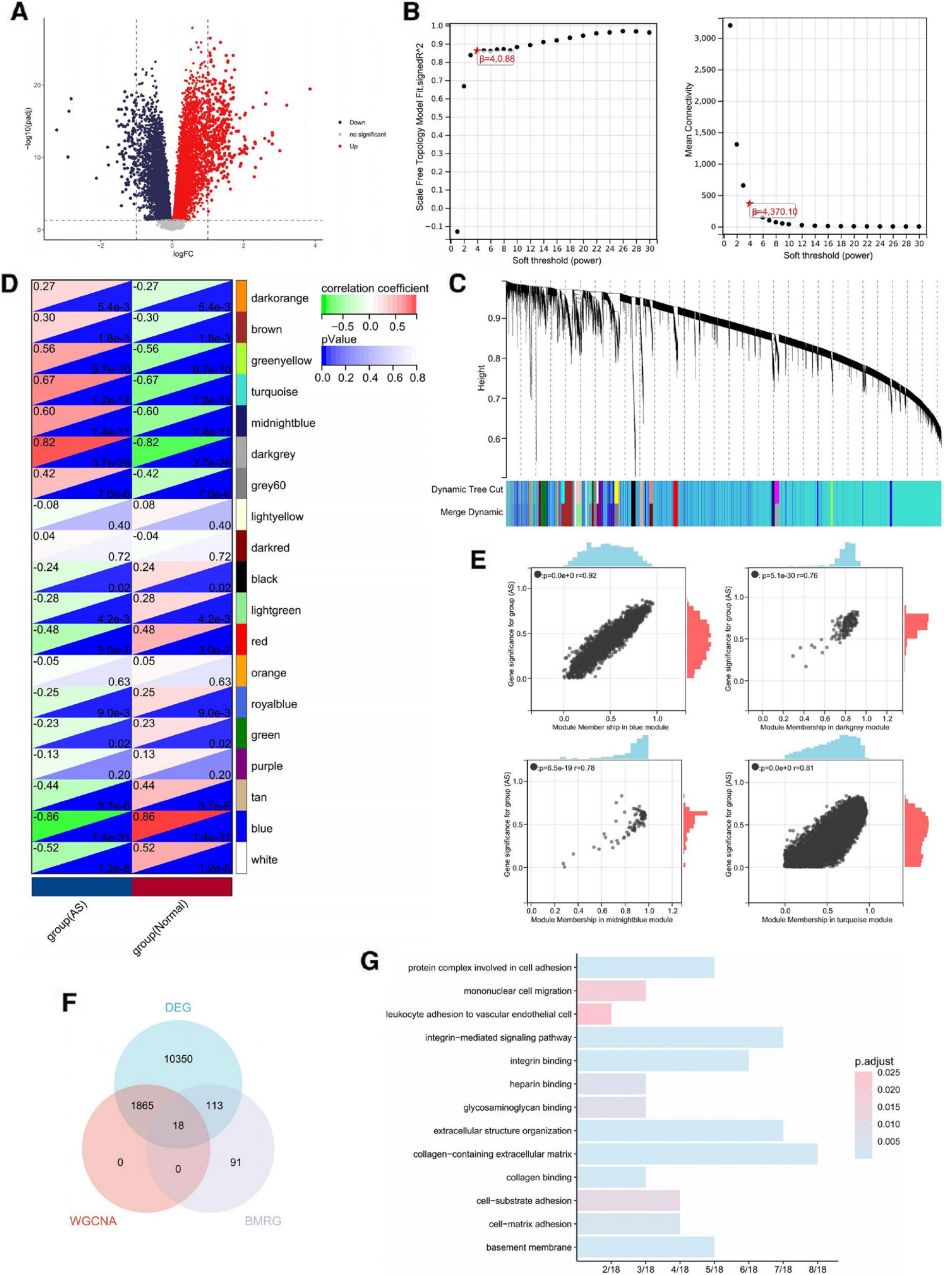
Identification of key basement membrane-related genes in AS. **A** Generation of a volcano plot of DEGs in AS, with genes having an adjusted *p* value less than 0.05 considered differentially expressed. **B** Determination of the optimal soft threshold β, set to 4. **C** Identification of co-expression modules in AS using the following parameters: minimum module size: 30; sensitivity: 3; module merge threshold: 0.25. **D** Correlation between the 19 identified co-expression modules and AS. Four modules (blue, dark gray, turquoise, and midnight blue) showed the highest AS correlation. **E** Scatter plots illustrating correlation between genes (module membership) in the top four modules and AS. **F** Intersection of DEGs, genes screened by WGCNA, and BMRGs in AS. **G** GO enrichment analysis of intersected genes in AS

The GSE100927 dataset, retrieved from the GEO database, was employed for AS analysis. It comprises 69 AS samples alongside 35 healthy controls. With the use of the limma package, 12,346 DEGs were identified, adhering to an adjusted *p* value threshold of less than 0.05. Notably, 6362 genes were significantly upregulated, and 5984 genes were correspondingly downregulated (Fig. 2A). WGCNA was conducted as previously described. Hierarchical clustering of samples illustrated that no sample diverged significantly as an outlier (Supplementary Fig. 2). The optimal soft threshold, β, was ascertained as 4 (Fig. 2B). A total of 19 co-expression modules were derived, with their correlations with AS illustrated (Fig. 2C, D). The top four modules displaying the greatest relevance to AS were singled out. The genes (module membership) within these four modules demonstrated a high degree of correlation with the disease, as shown in Fig. 2E. Employing a threshold of an MM-GS correlation coefficient greater than 0.8, 1883 genes were singled out from these four modules (Supplementary Table 3). An intersection of DEGs, WGCNA-screened genes, and BMRGs identified 18 key basement membrane-related genes (Fig. 2F). GO enrichment analysis indicated that these genes have substantial involvement in cell adhesion and migration processes, specifically in mononuclear cells (Fig. 2G).

### Identification of *ITGAM* as a crucial gene through machine learning screening

**Fig. 3:**
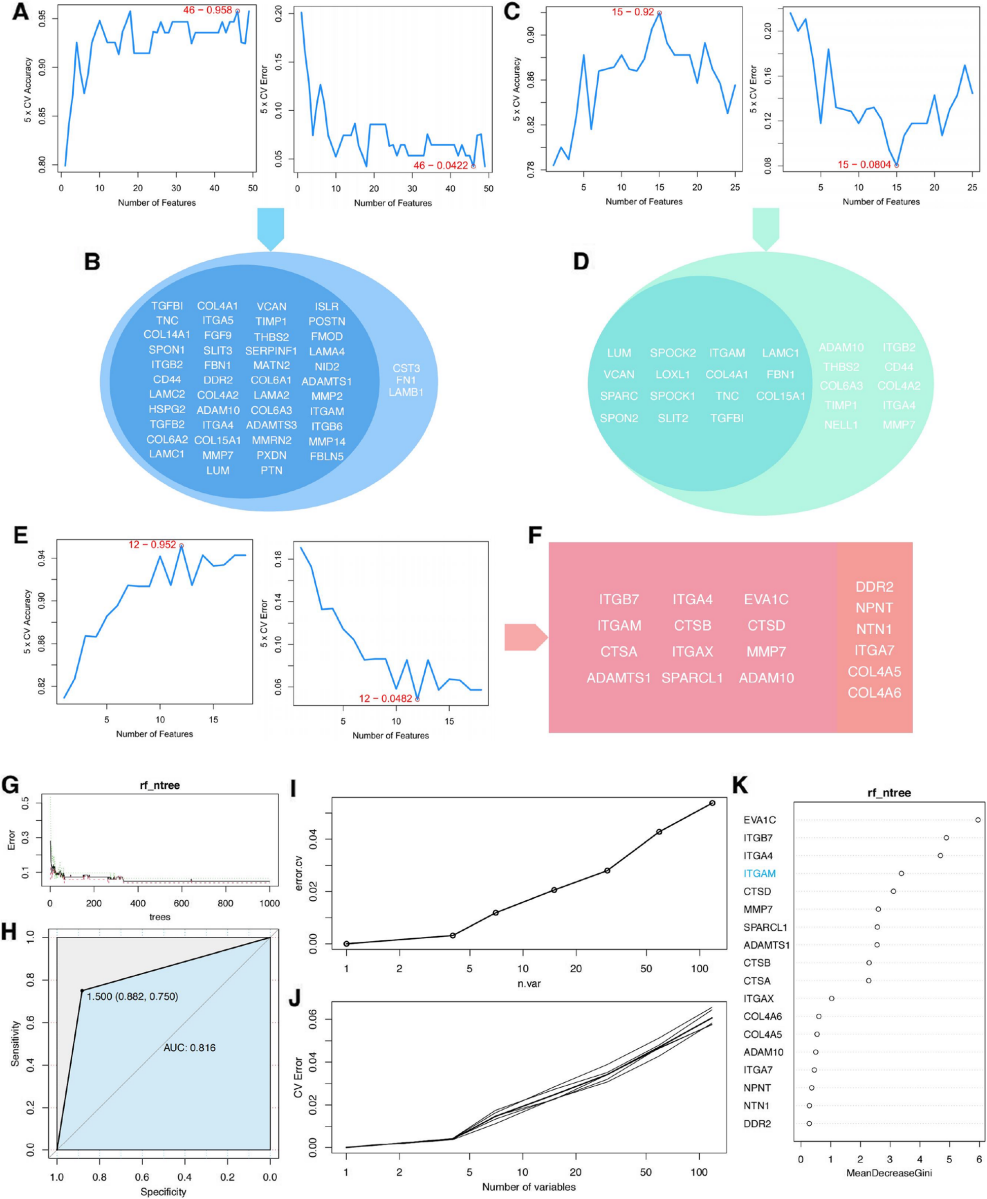
Identification of *ITGAM* as a crucial gene using machine learning algorithms. **A**, **C**, **E** SVM-RFE model construction for the key genes in DN glomerulus, DN tubulointerstitium, and AS, respectively. The results of a five-fold cross-validation are displayed. CV: cross-validation. **B**, **D**, **F** The left side presents the genes endorsed by the SVM-RFE model; the right side displays the excluded genes. **G** Experimental training model of RF. (H) ROC curve analysis was conducted to evaluate the predictive performance of the model. AUC = 0.816. **I** Results of single-fold cross-validation. **J** Results of five-fold cross-validation. **K** Importance ranking of the 18 hub genes in AS, based on the mean decrease in the Gini index

Our study aimed to identify key basement membrane-related genes in the context of DN glomerulus, DN tubulointerstitium, and AS pathologies. We applied traditional machine learning algorithms and employed SVM-RFE to screen these contexts’ associated hub genes. In DN glomerulus, five-fold cross-validation results suggested that the optimal selection comprised the top 46 genes from the SVM-RFE ranking within the 49 hub genes. This selection resulted in the lowest error and highest accuracy rates (Fig. 3A, B). For DN tubulointerstitium, the SVM-RFE model recommended selecting the top 15 hub genes (Fig. 3C, D). Similarly, for AS, the SVM-RFE model proposed selecting the top 12 hub genes (Fig. 3E, F). From these selections, only *ITGAM* was consistently identified as a key gene across DN glomerulus, DN tubulointerstitium, and AS. To further validate the significance of *ITGAM* in AS, we constructed an RF model. In the preliminary training phase, we set the tree count to 1000. The prediction model achieved stability at approximately 640 trees (Fig. 3G). In the formal training phase, we selected 750 trees. The ROC curves demonstrated satisfactory prediction accuracy for this model (Fig. 3H). Further, both single and five-fold cross-validations were performed. We observed a marked increase in the model’s error rate when the variable count exceeded 4 (Fig. 3I, J). The RF algorithm ranked the importance of 18 hub genes in AS based on the mean decrease in the Gini index. The top four genes were *EVA1C*, *ITGB7*, *ITGA4*, and *ITGAM*, with *ITGAM* being one of the most crucial (Fig. 3G). In conclusion, the findings from our comprehensive analysis indicate that *ITGAM* likely plays a significant role in basement membrane lesions within DN glomerulus, DN tubulointerstitium, and AS.

### *ITGAM* is significantly associated with tissue immune infiltration in DN and AS

**Fig. 4:**
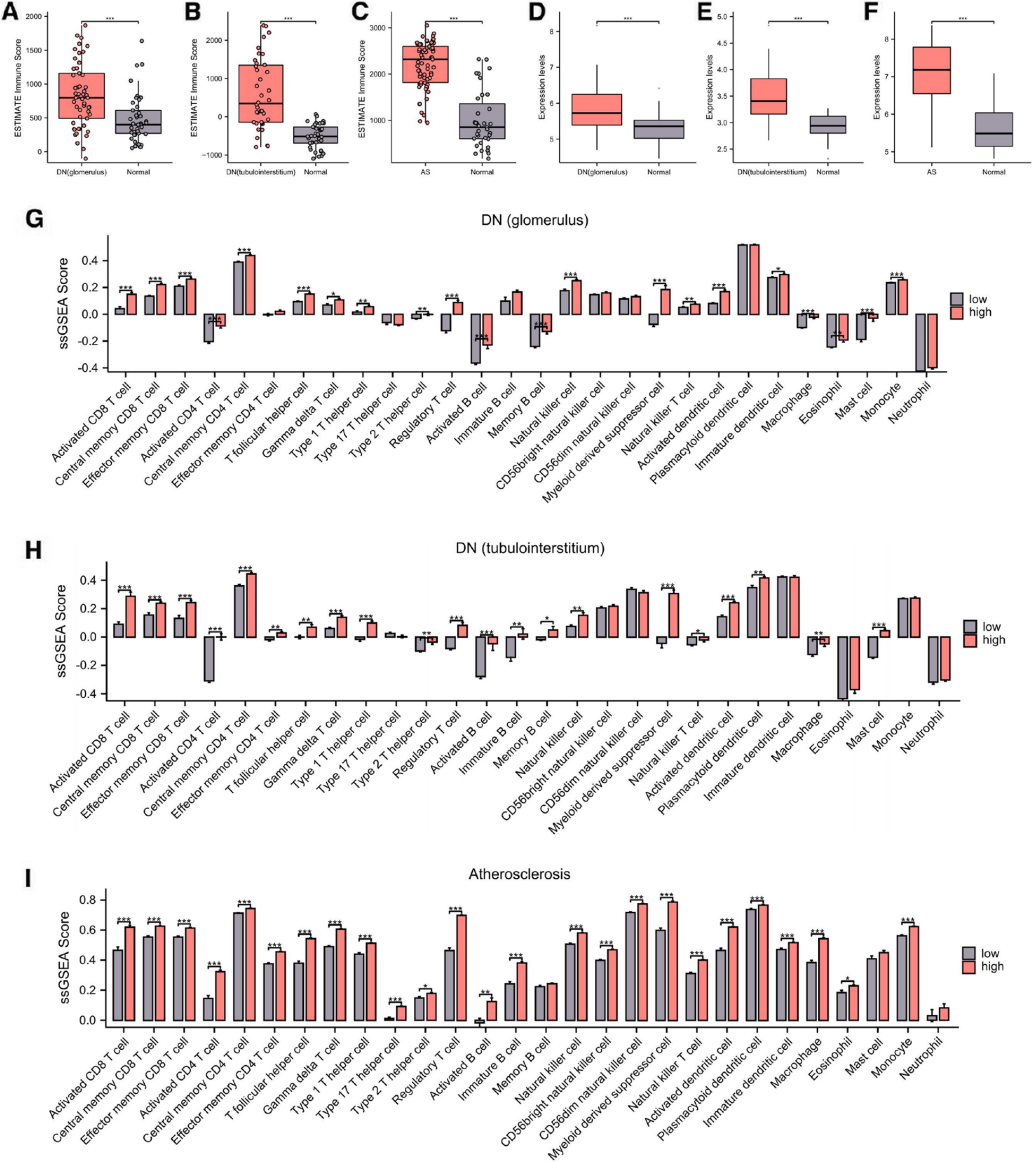
Analysis of *ITGAM*-related immune infiltration. **A**, **B**, **C** Overall immune infiltration scores for DN glomerulus, DN tubulointerstitium, and AS, calculated using the ESTIMATE algorithm. **D**, **E**, **F** Expression levels of ITGAM in DN glomerulus, DN tubulointerstitium, and AS. **G**, **H**, **I** Infiltration levels of 28 types of immune cells in ITGAM high-expression and low-expression groups for DN glomerulus, DN tubulointerstitium, and AS, assessed using ssGSEA

Our initial assessment was focused on the overall immune infiltration in three distinct tissues: DN glomerulus, DN tubulointerstitium, and AS, employing the ESTIMATE algorithm. Immune infiltration was substantially elevated in all these three tissues relative to normal tissue controls (Fig. 4A-C). These findings indicate an active involvement of the immune system in the pathogenesis of both diabetic nephropathy and atherosclerosis. Our investigation further revealed a significant upregulation of *ITGAM*, an integrin subunit, in these aforementioned lesioned tissues (Fig. 4D-F). To elucidate the connection between *ITGAM* expression levels and immune infiltration, we sorted the DN glomerulus, DN tubulointerstitium, and AS samples in descending order based on *ITGAM* expression levels. The top 20% of samples were categorized as the high-expression group and the bottom 20% as the low-expression group. We then implemented the ssGSEA to ascertain the infiltration levels of 22 distinct immunocyte types. Our data revealed that the infiltration levels of immunocytes were markedly higher in the *ITGAM* high-expression group for all three lesioned tissues under consideration (Fig. 4G-I). This observation underscores a significant association between *ITGAM* expression and tissue immune infiltration in both DN and AS.

### Influence of *ITGAM* expression levels on basement membrane component-encoding genes

**Fig. 5:**
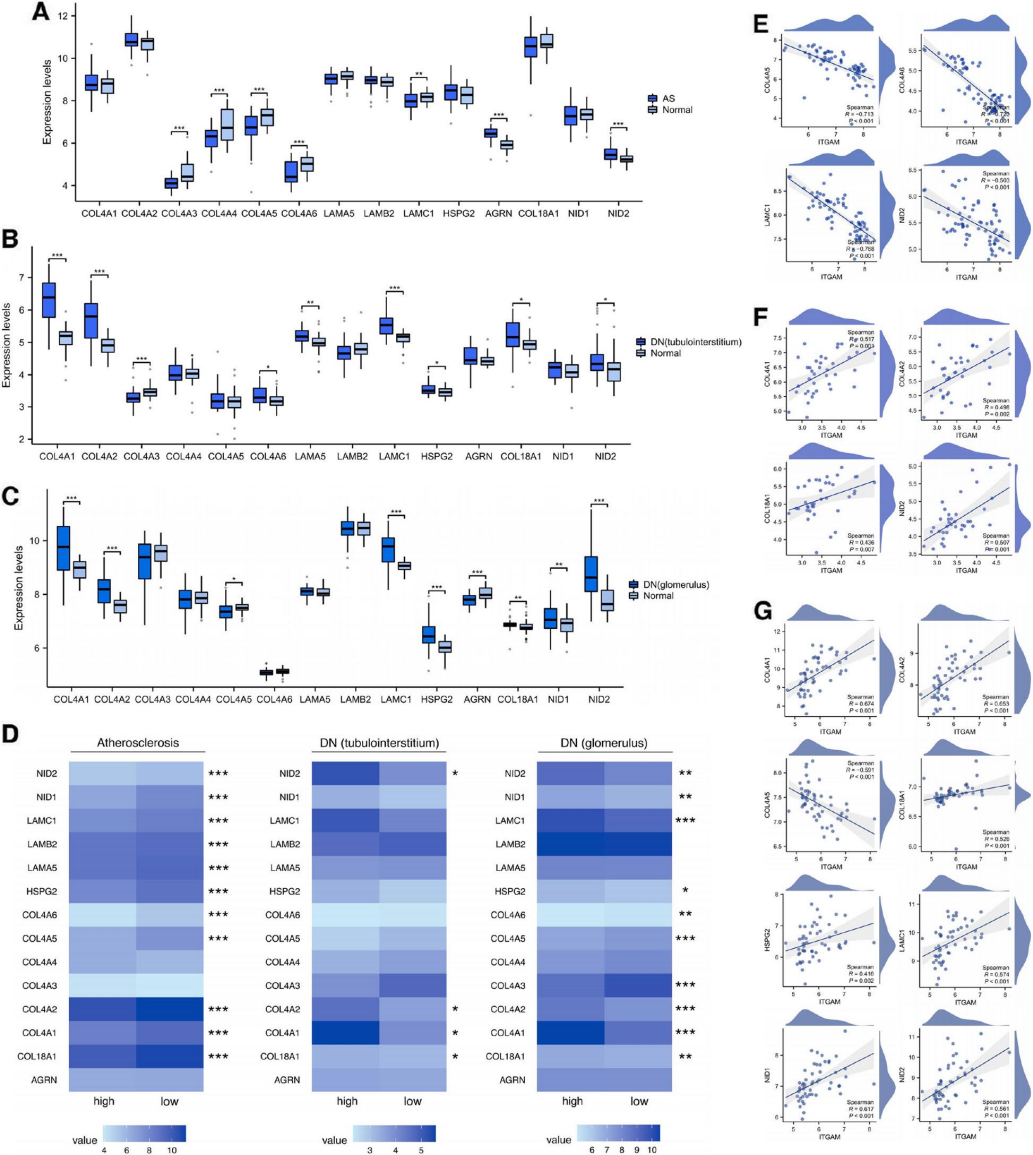
*ITGAM* influences the expression of genes encoding major components of the basement membrane. **A** Comparison of expression levels of genes encoding major basement membrane components between AS and normal tissues. **B** Comparison of expression levels of genes encoding major basement membrane components between DN tubulointerstitium and normal control. **C** Comparison of expression levels of genes encoding major basement membrane components between DN glomerulus and normal control. **D** Heatmap representing the expression levels of genes encoding major components of the basement membrane in ITGAM high-expression and low-expression groups in DN glomerulus, DN tubulointerstitium, and AS. **E** Correlation analysis of *COL4A5*, *COL4A6*, *LAMC1*, and *NID2* with *ITGAM* in DN glomerulus. **F** Correlation analysis of *COL4A1*, *COL4A2*, *COL18A1*, *and NID2* with *ITGAM* in DN tubulointerstitium. **G** Correlation analysis of *COL4A1*, *COL4A2*, *LAMC1*, *HSPG2*, *COL18A1*, *NID1*, and *NID2* with *ITGAM* in AS

Our study encompassed an investigation into the expression of genes encoding key constituents of the GBM, including COL4A1, COL4A2, COL4A3, COL4A4, COL4A5, COL4A6, LAMA5, LAMB2, LAMC1, HSPG2, AGRN, COL18A1, NID1, and NID2 [25]. These elements are also pivotal in the formation of arterial basement membranes and TBM. In the context of AS, we observed downregulation of *COL4A3*, *COL4A4*, *COL4A5*, *COL4A6*, and *LAMC1*, contrasted by upregulation of *AGRN* and *NID2* (Fig. 5A). For DN tubulointerstitium, upregulated genes comprised *COL4A1*, *COL4A2*, *COL4A6*, *LAMA5*, *LAMC1*, *HSPG2*, *COL18A1*, and *NID2*, while *COL4A3* alone was downregulated (Fig. 5B). The DN glomeruli displayed upregulated expression of *COL4A1*, *COL4A2*, *LAMC1*, *HSPG2*, *COL18A1*, *NID1*, and *NID2*, counteracted by downregulation of *COL4A5* and *AGRN* (Fig. 5C). We further divided our sample into *ITGAM* high-expression and low-expression groups, employing the same methodology. Comparison of gene expression levels for major basement membrane components between these groups was performed across the three types of pathological tissues (Fig. 5D). In atherosclerotic tissues, we noted differential expression of *COL4A5*, *COL4A6*, *LAMC1*, and *NID2* compared to control tissues, along with distinct expression levels in the high and low *ITGAM* expression groups. Spearman’s correlation analysis conducted for these genes with *ITGAM* unveiled consistent negative correlations (Fig. 5E). In the DN tubulointerstitium, there was significant differential expression of *COL4A1*, *COL4A2*, *COL18A1*, and *NID2* compared to control tissues, and between the *ITGAM* high- and low-expression groups. These genes demonstrated substantial positive correlations with *ITGAM* (Fig. 5F). In the DN glomerulus, we found that *COL4A1*, *COL4A2*, *LAMC1*, *HSPG2*, *COL18A1*, *NID1*, and *NID2* all displayed positive correlations with *ITGAM*, while *COL4A5* showed a negative correlation with *ITGAM* (Fig. 5G). The aggregate of our findings suggests that *ITGAM* expression levels modulate the expression of genes responsible for encoding key components of the basement membrane.

### ITGAM is predominantly expressed on the surface of macrophages in DN and AS

**Fig. 6:**
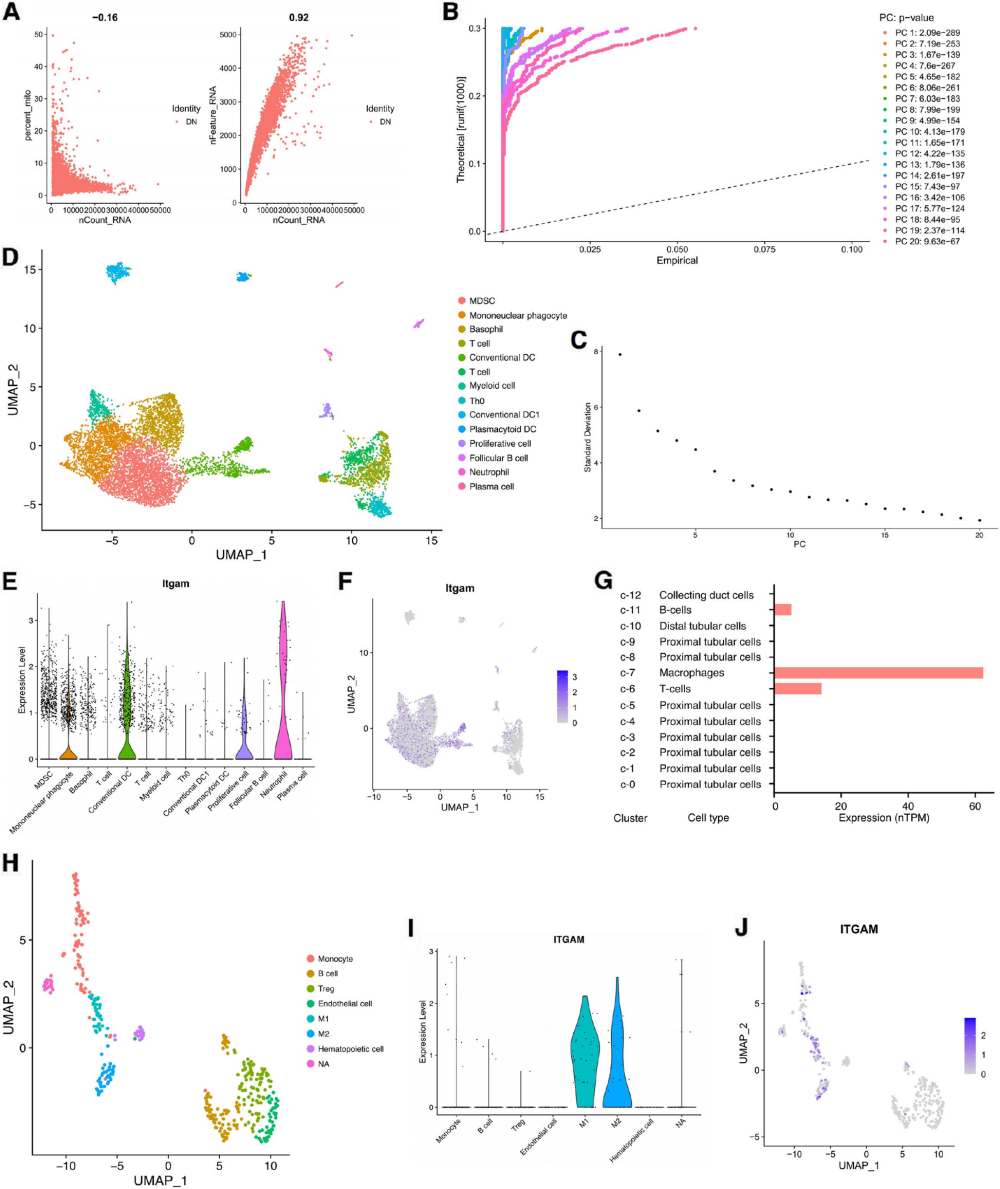
Single-cell analysis of DN and AS samples. **A** A correlation analysis depicting the relationship between RNA quantity, the proportion of mitochondrial genes, and the total number of genes. **B** JackStrawPlot was used to assess the significance of the principal components. **C** ElbowPlot was utilized to determine the optimal number of principal components. **D** UMAP visualizing the distinct cell subpopulations within the DN sample. **E** Violin plot showcasing *ITGAM* expression levels across different cell subpopulations. **F** UMAP demonstrating *ITGAM* expression across different cell subpopulations. **G** The expression levels of *ITGAM* across various cell types in the kidney, as sourced from the Human Protein Atlas database. **H** UMAP of single-cell analysis visualizing cell subpopulations within the AS sample. **I** Violin plot of *ITGAM* expression levels in each cell subpopulation. **J** UMAP showing the expression of *ITGAM* in different cell subpopulations

The differential expression of *ITGAM* across various cell types within DN and AS tissues was evaluated using single-cell sequencing analysis. We employed sample GSM5851040 (derived from diabetic OVE26 mice) from the GSE195799 dataset for DN-specific single-cell examination. A preliminary evaluation of the number of expressed genes, RNA quantity, and mitochondrial gene proportion was carried out across multiple cells and samples (Supplementary Fig. 3A). We observed a negligible correlation between the RNA quantity and the proportion of mitochondrial genes, while a strong positive correlation was identified between the number of expressed genes and RNA quantity (Fig. 6A). This indicated that the cells within the samples were normal and fit for analysis. We then refined the analysis by excluding cells with fewer than 200 or more than 3000 transcripts, or with a mitochondrial gene proportion exceeding 5%. The threshold for highly variable genes was set to 2500 (Supplementary Fig. 3B). Dimension reduction was performed using principal component analysis (PCA), selecting 14 principal components (Supplementary Fig. 3C, Fig. 6B, C). This enabled us to categorize the cells into 14 distinct subpopulations, utilizing the Cell Marker 2.0 database for annotations (http://www.bio-bigdata.center) [26] (Fig. 6D). Notably, we found a preponderance of *ITGAM* expression in mononuclear phagocytes, conventional dendritic cells, proliferating cells, and neutrophils (Fig. 6E, F). Complementing this, data from the Human Protein Atlas (https://www.proteinatlas.org/) revealed a primary ITGAM expression in kidney macrophages [27] (Fig. 6G). For AS, we carried out a similar single-cell analysis using the GSM5577200 sample from the GSE184073 dataset (Supplementary Fig. 3D, E). We excluded cells with fewer than 200 or over 4000 transcripts, or those having a mitochondrial gene proportion beyond 5%. We set the highly variable gene threshold to 5000 for this analysis (Supplementary Fig. 3F). Post-PCA dimension reduction, the principal components were confined to 10, allowing the classification of cells into eight subpopulations (Supplementary Fig. 3G-I, Fig. 6I). In alignment with the DN findings, *ITGAM* was majorly expressed in macrophages, specifically within the M1 and M2 phenotypes (Fig. 6I, J). We further analyzed the proteomics of human monocytes in AS related and non-related to CKD, accessed through the ProteomeXchange database. These results demonstrated that the abundance of ITGAM is significantly higher in the early stages of CKD-related AS compared to simple AS, which might indicate that ITGAM is involved in the common underlying pathology of DN and AS (Supplementary Fig. 3J).

## Discussion

Diabetic nephropathy, a common microvascular complication of diabetes, is often difficult to diagnose at its early stages. By the time clinical confirmation is achieved, disease progression is typically advanced and challenging to manage, often culminating in ESRD. Current clinical practice mainly relies on symptomatic treatment, and kidney replacement therapy or transplantation are usually the selected options when patients progress to ESRD. The presence of microalbuminuria often signifies substantial renal structural damage [5]. A plethora of research has highlighted the role of podocyte injury, characterized by a reduction in podocyte count/density and foot process fusion/effacement, in DN progression. This injury is viewed as a significant contributor to the development of albuminuria [28–30]. Nevertheless, the mechanisms through which basement membrane thickening leads to albuminuria are not yet fully understood. Several hypotheses suggest that modifications in GBM components, such as increased type IV collagen and decreased heparan sulfate proteoglycans (HSPG), cause alterations in the charge barrier and filtration slits of GBM. These changes may stimulate the onset and progression of proteinuria [5, 31]. Thickening of the TBM and extracellular matrix accumulation in the kidney may also play a role in compromised protein reabsorption. Thus, preventing GBM thickening could be a viable therapeutic strategy for early intervention in DN progression. Podocytes are widely acknowledged to play a role in balancing the synthesis and degradation of GBM. Alterations in podocyte function in DN lead to GBM remodeling and thickening. Some theories suggest that GBM thickening is a compensatory response to podocyte injury, aimed at mitigating proteinuria. This phenomenon may elucidate why GBM thickening transpires before the onset of proteinuria and could potentially be one of the causes of non-proteinuric DN (NP-DN). In most cases, however, this compensatory change rapidly progresses to decompensation, initiating a destructive cycle of GBM disorganization and barrier dysfunction [5]. Despite these insights, the pathophysiological mechanisms underpinning basement membrane thickening remain elusive.

ESRD represents the most severe manifestation of DN, but clinical observations suggest that cardiovascular diseases, not the necessity for renal replacement therapy, account for the majority of patient mortality [2]. This is primarily attributed to the damage and degradation of the atherosclerotic plaque’s basement membrane, which in turn accelerates plaque formation. Our research involved performing WGCNA on 222 BMRGs. The goal was to identify pivotal genes in DN glomerulus, DN tubulointerstitium, and AS. It is known that cells communicate with the extracellular matrix via cell surface receptors, particularly integrins. These receptors are critical for cell adhesion and signal transduction from the extracellular matrix to the cell. *ITGAM*, a gene that encodes the integrin alpha M chain (also referred to as CD11b), facilitates this communication by adhering to the basement membrane. This promotes leukocyte recruitment to inflammatory sites. In the context of lupus nephritis, ITGAM is thought to regulate negatively the activities of innate immune cells such as macrophages and neutrophils within pro-inflammatory signaling pathways [32]. Therefore, ITGAM-targeting agonists represent a potential treatment for lupus nephritis. However, the specific role of ITGAM in DN remains largely unexplored. A robust immune response and inflammation infiltration are common characteristics of both DN and AS. Using the ESTIMATE algorithm, we confirmed this trend across three sample groups. Additionally, our ssGSEA analysis revealed that elevated *ITGAM* expression is positively correlated with immunocytes infiltration in the afflicted tissues. Single-cell analysis also disclosed the predominant expression of *ITGAM* in macrophages within DN and AS tissues.

Aberrant glucose and lipid metabolism in DN triggers substantial macrophage accumulation in the glomerulus and tubulointerstitium [33–36]. High glucose levels can stimulate macrophages to release TNF-α, which can induce podocyte apoptosis. Moreover, macrophages can trigger podocyte pyroptosis and autophagy via the secretion of exosomes containing miRNA. They can also interact with mesangial cells to stimulate extracellular matrix secretion [37, 38]. Persistent chronic inflammation can further lead to glomerulosclerosis and tubulointerstitial fibrosis via macrophage-myofibroblast transition (MMT) [39]. However, the role of macrophages in DN basement membrane thickening remains unclear. Despite this uncertainty, it is unequivocal that macrophages contribute significantly to atherosclerotic plaque formation. We therefore conducted further analyses to explore the relationship between *ITGAM* expression levels and the genes encoding the basement membrane’s major components. Our findings revealed a strong correlation between elevated ITGAM expression and dysregulated expression of basement membrane-associated genes. Our analysis suggests a mechanism in which macrophages adhere to the GBM and TBM via ITGAM. This leads to basement membrane thickening and remodeling in DN. Similarly, in AS, macrophage migration to lesions and adherence to the basement membrane through ITGAM might result in basement membrane injury and degradation. Macrophages’ capacity to secrete matrix metalloproteinases (MMP), which degrade collagen, might partially explain their contribution to basement membrane injury and remodeling [40]. MMP-inhibitors have been demonstrated to confer significant disease-protective effect in the early stages of Alport Syndrome (before the onset of proteinuria), through the maintenance of GBM/extracellular matrix integrity [41].

Our research tentatively suggests the possibility of a shared mechanistic underpinning for basement membrane degradation in both DN and AS. Accordingly, we propose the hypothesis that modulating ITGAM could offer a potentially innovative therapeutic approach for early-stage DN intervention and prevention of concomitant AS. It is important to acknowledge that our current research is confined to data analysis, which confirmed a strong correlation between ITGAM and basement membrane lesions in DN and AS, but did not establish a causal relationship. Additional mechanistic studies are required to further elucidate the precise role of ITGAM and macrophages in the pathophysiological process of basement membrane damage in DN and AS.

### Supplementary Information

**Supplementary material 1.**

## Data Availability

The datasets analyzed during the current study are available in the Gene Expression Omnibus (GEO) database (https://www.ncbi.nlm.nih.gov/geo/) and the ProteomeXchange database (https://proteomecentral.proteomexchange.org/). GSE96804: https://www.ncbi.nlm.nih.gov/geo/query/acc.cgi?acc=GSE96804 GSE104948: https://www.ncbi.nlm.nih.gov/geo/query/acc.cgi?acc=GSE104948 GSE104954: https://www.ncbi.nlm.nih.gov/geo/query/acc.cgi?acc=GSE104954 GSE30529: https://www.ncbi.nlm.nih.gov/geo/query/acc.cgi?acc=GSE30529 GSE47184: https://www.ncbi.nlm.nih.gov/geo/query/acc.cgi?acc=GSE47184 GSE100927: https://www.ncbi.nlm.nih.gov/geo/query/acc.cgi?acc=GSE100927 GSE195799: https://www.ncbi.nlm.nih.gov/geo/query/acc.cgi?acc=GSE195799 GSE184073: https://www.ncbi.nlm.nih.gov/geo/query/acc.cgi?acc=GSE184073 PXD041367:https://proteomecentral.proteomexchange.org/cgi/GetDataset?ID=PXD041367 The data generated during the current study are available from the corresponding author on reasonable request.
