# Supplementary material for: ITGAM-mediated macrophages contribute to basement membrane damage in diabetic nephropathy and atherosclerosis
Source: BMC Nephrol. 2024 Feb 27;25:72. doi: 10.1186/s12882-024-03505-1 (PMC10900706; doi:10.1186/s12882-024-03505-1)
Supplement: Supplementary file 1 — Supplementary material 1. [file 12882_2024_3505_MOESM1_ESM.zip › supplement/Supplementary information.docx]

**Supplementary information**

Macrophages are involved in basement membrane damage in diabetic nephropathy and atherosclerosis via ITGAM

**Supplementary Figure 1.** Supplement to WGCNA of DN tubulointerstitium. (A) Using the gene expression profile of GSE104954, GSE30529, and GSE47184, hierarchical clustering of samples was performed based on expression levels. (B, C) Screening and determination of the optimal soft threshold β (β was set to 12). (D) Identification of co-expression modules in DN tubulointerstitium. The minimum module size: 30; sensitivity: 3; module merge threshold: 0.25. (E) The correlation between the 13 co-expressed modules identified and DN (tubulointerstitial group). Pink, darkorange, lightcyan, and darkgreen were the four modules with the highest correlation to DN. (F-I) Scatter plots of the correlation between genes (module membership) in the top four modules and DN (tubulointerstitial group).

**Supplementary Figure 2.** Using the gene expression profile of GSE100927, hierarchical clustering of samples was performed based on expression levels.


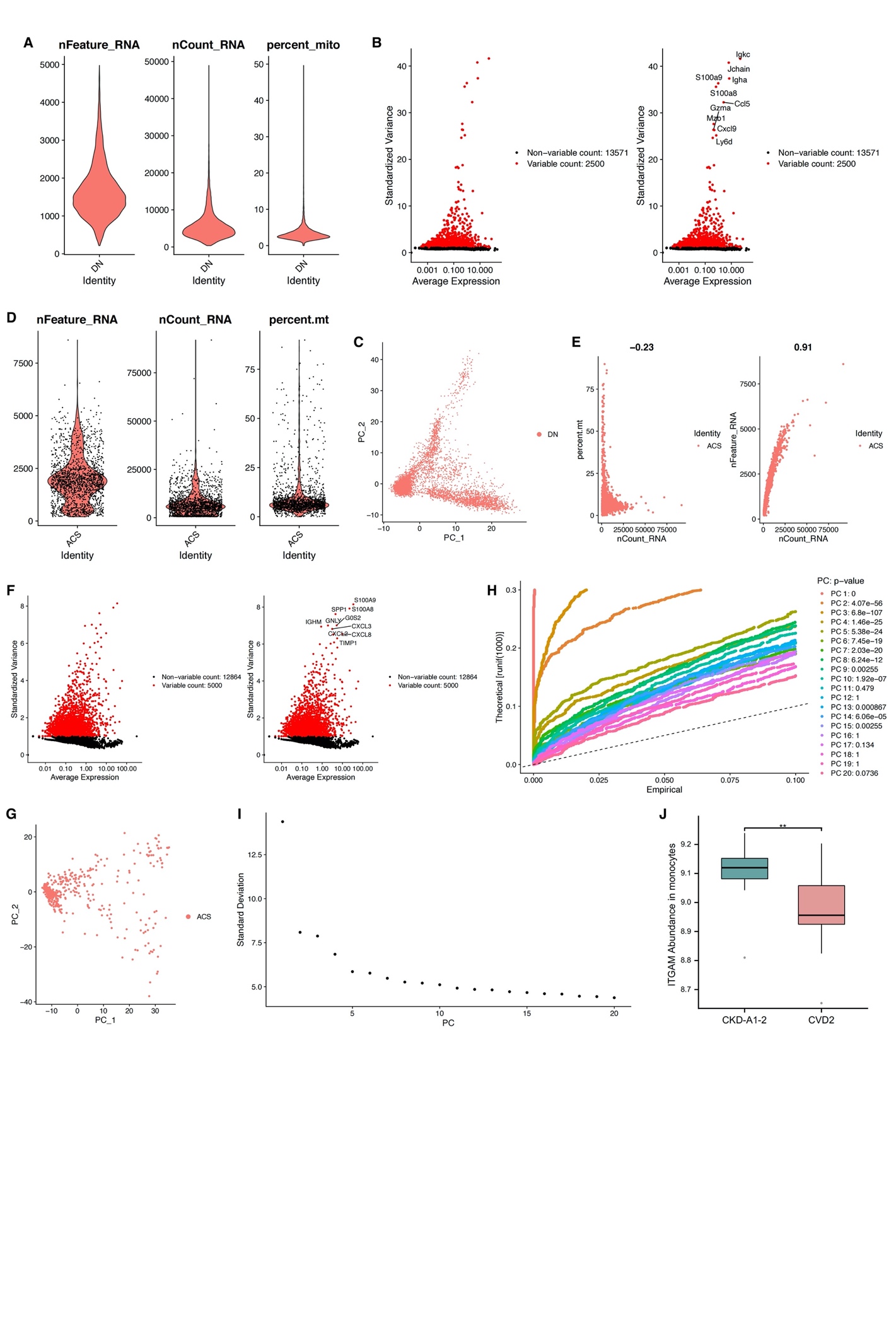


**Supplementary Figure 3. Single-cell analysis of DN and AS samples.** (A) The number of genes expressed in different samples of DN, and the quantity of RNA and the percentage of mitochondrial genes in different cells. (B) Volcano plot of highly variable genes in GSM5851040. (C) PCA for dimension reduction of DN sample. (D) The number of genes expressed in different samples of acute coronary syndrome (ACS), and the quantity of RNA and the percentage of mitochondrial genes in different cells. (E) Correlation between the quantity of RNA, the percentage of mitochondrial genes and the number of genes. (F) Volcano plot of highly variable genes in GSM5577200. (G) PCA for dimension reduction of ACS sample. (H) JackStrawPlot was performed for determination of the dimensions of the principal components. (G) ElbowPlot was generated to determine the the dimensions of the principal components. (J) Proteomic analysis of human monocytes in AS related and non-related to CKD. The abundance of ITGAM is significantly higher in stages 1 to 2 of AS related to CKD (CKD-A1-2) compared to AS non-related to CKD (CVD2).
